# Analysis of memory T lymphocyte activity following stimulation with overlapping HLA-A*2402, A*0101 and Cw*0402 restricted CMV pp65 peptides

**DOI:** 10.1186/1479-5876-3-23

**Published:** 2005-05-26

**Authors:** Monica Ghei, David F Stroncek, Maurizio Provenzano

**Affiliations:** 1 Washington University School of Medicine, St. Louis, MO, USA; 2 Molecular Immunology Sections, Department of Transfusion Medicine, National Institutes of Health, Bethesda, MD, USA; 3 Institut für chirurgische Forschung und Spitalmanagement, University of Basel, Switzerland

## Abstract

The continuous efforts aimed at the identification of new immune epitopes across the MHC system has led to the discovery that more than one peptide may be restricted to the same HLA antigen and function as an immune determinant for that association. The aim of this study was to compare the ability of two overlapping peptides, the nonamer (9-mer) cytomegalovirus (CMV) pp65_341–349 _(QYDPVAALF) and the decamer (10-mer) CMV pp65_341–350 _(QYDPVAALFF), and the esadecamer (16-mer) peptide containing both the 9-mer and 10-mer sequences, CMV pp65_340–355 _(RQYDPVAALFFFDIDL), to stimulate and maintain over time a T cell immune reactivation by HLA-A*2402, A*0101, and Cw*0402 cells from CMV-seropositive subjects. The 9-mer, 10-mer, and 16-mer peptides effectively stimulated CTLs from HLA-A*2402, HLA-A*0101, and HLA-Cw*0402 CMV seropositive donors. This data confirms that both the 9-mer and the 10-mer peptides are promiscuous and are not restricted to a single HLA antigen. CMV pp65_341–349 _and CMV pp65_341–350 _have the ability to produce CMV-specific CTLs in subjects with several different HLA types, presenting a practical advantage over other peptides that are restricted only to a single HLA antigen, and thus being optimal for CMV adoptive immune therapy. Moreover, since the 16-mer peptide encompasses both the 9-mer and 10-mer peptides, it may be better than either of these peptides for CMV adoptive immune therapy.

## Introduction

In healthy subjects, primary infection with Cytomegalovirus (CMV) is usually mild or asymptomatic and is effectively controlled by the cell-mediated immune response [1]. However, in immune compromised individuals, such as those with AIDS or after bone marrow transplantation, CMV reactivation is associated with significant morbidity until the individual's immune system is completely reconstituted [2]. One method of preventing post-transplant CMV infection is adoptive immunotherapy using CMV-specific cytotoxic T cells (CTLs) from the transplant donor [3]. Several HLA class I restricted peptides derived from the immune dominant CMV 65 kd matrix phosphoprotein (pp65) have been shown to produce CMV-specific CTLs. Two overlapping HLA-A*2402 restricted peptides have been described: pp65_341–349 _and pp65_341–350_. These peptides are a nonamer (9-mer) and a decamer (10-mer) that overlap except for the last amino acid phenylalanine (F) at the C-terminus [QYDPVAALF(F)]. Despite their similarity, the ability of these peptides to induce a T cell response has been reported to differ [4,5].

Although it has been generally accepted that a unique CMV peptide is bound and presented by each separate HLA class I molecule, recent studies suggest that certain peptides are more promiscuous and may be presented by more than one HLA class I antigen. In this specific case, the 9-mer pp65_341–349 _has been shown to stimulate CTLs from both HLA-A*2402 and Cw*0402 donors [6], while the 10-mer pp65_341–350 _has been shown to be reactive with both HLA-A*2402 and A*0101 donors [7].

The current investigation sought to compare the potency of these two peptides and determine the optimum peptide size for effective CMV adoptive immune therapy. Both peptides were tested for their ability to stimulate CMV-specific CTLs in HLA-A*2402, HLA-A*0101, and HLA-Cw*0402 restriction. In addition, a 16-mer pp65_340–355 _that includes both the 9-mer and the 10-mer peptides was tested for its ability to reactivate memory T cells. This specific 16-mer peptide was selected since it represents the naturally processed peptide that would encompass both the 9-mer and 10-mer peptides. IFN-γ mRNA transcript production was measured by *in vitro *cell culture assays in which the cells were peptide-induced for 3 hours after a 2-week *in vitro *sensitization, while IFN-γ protein release was measured using *in vitro *cell culture supernatant collected at different time points.

The goal of the investigation was to determine whether both the 9-mer and the 10-mer peptides maintain high levels of CTL stimulation over time for all HLA restrictions studied. Moreover, it was important to investigate whether stimulation with the naturally processed 16-mer peptide, followed by re-stimulation by the two smaller peptides embedded within the larger sequence, lead to effective T cell memory immune response.

## Materials and methods

### Peptide selection, synthesis and nomenclature

**Figure 1:**
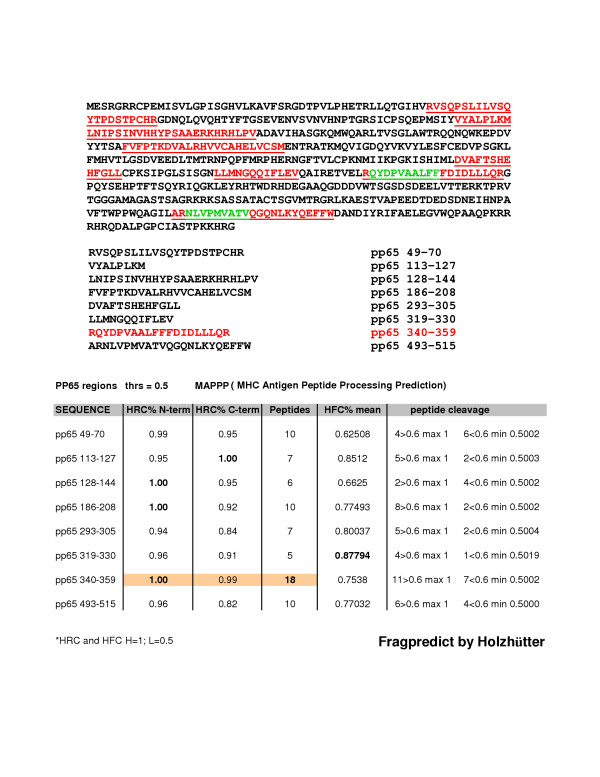
**Scheme of candidate immune regions within the CMV pp65 as analyzed by MAPP. **The immune dominant CMV 65 kd matrix phosphoprotein (pp65) is shown above. The highlighted red sequences within the pp65 represent the most immunogeneic polypeptides, as predicted by the MAPPP algorithm (threshold ≤ 0.5). Their positions in the protein are listed below. The two highlighted green peptides, NLVPMVATV and QYDPVAALF(F) are, respectively, the known HLA-A*0201 associated pp65_495–503 _and the two HLA-A*2402 associated overlapping peptides pp65_341–349 _and pp65_341–350_. The table refers to each single polypeptide's degree of cleavage by proteasome activity at either N-terminus or C-terminus. The number of peptides derived from each sequence's proteolitic activity and the corresponding degree of peptide cleavage are also shown. HRC (Highest residue cleavage probability); HFC (Highest fragment cleavage probability).

Two overlapping peptides derived from the immune dominant CMV 65 kd matrix phosphoprotein (pp65), the nonamer pp65_341–349 _(QYDPVAALF) and the decamer pp65_341–350 _(QYDPVAALFF) peptides were used to analyze memory T lymphocyte activity in PBMCs collected from CMV seropositive donors bearing alleles HLA-A*2402, A*0101, or Cw*0402. The esadecamer pp65_340–355 _(RQYDPVAALFFFDIDL) sequence that encompasses both pp65_341–349 _(QYDPVAALF) and pp65_341–350 _(QYDPVAALFF) peptides was selected according to its score, as analyzed by MAPPP (MHC Antigen Peptide Processing Prediction) [8] based on FRAGPREDICT developed by Holzhütter [9] (Figure 1). The original sequence of 20 amino acids in length (CMV pp65_340–359_), as shown in figure 1, was reduced to 16 amino acids (CMV pp65_340–355_) in order to allow the complete reconstitution of this sequence. The C-terminus of the peptide was reduced to form a 16-mer sequence and the selection of the 16-mer did not affect the aim of having both 9-mer and 10-mer peptides represented in the sequence. The HLA restriction for this 16 amino acid sequence was not known (Additional File 1).

The three peptides were synthesized by Princeton Biomolecules (Langhorne, PA) with purity from 90% to 100% as analyzed by High Performance Liquid Chromatography (HPLC), dissolved at 100 μg/ml in 50% DMSO and stored at 4°C. To simplify the peptide nomenclature in this paper we refer to peptide CMV pp65_341–349 _(QYDPVAALF) as the 9-mer peptide, to peptide CMV pp65_341–350 _(QYDPVAALFF) as the 10-mer peptide, and to sequence CMV pp65_340–355 _(RQYDPVAALFFFDIDL) as the 16-mer peptide.

### Donor collection and cell preparation

Leukocytes were collected from CMV seropositive donors bearing alleles HLA-A*2402, A*0101, or Cw*0402 after obtaining informed consent. The presence of CMV antibodies was analyzed by passive latex agglutination (CMVSCAN kit, Becton Dickinson Microbiology System, Cockeysville, MD). MHC class I genotypes were determined by sequence-specific primer polymerase chain reaction (PCR). Lymphapheresis was performed using a CS3000 Plus blood cell separator (Fenwal Divison, Baxter Health Care, Deerfield, IL), and PBMCs were isolated from the apheresis product by Ficoll (Pharmacia Biotech, Wilkstrom, Sweden) density gradient centrifugation.

### PBMCs *in vitro *sensitization

The *in vitro *sensitization involved a 2-week cell culture in the presence of peptide and IL-2. Briefly, a 2-week *in vitro *sensitization was performed using PBMCs from CMV-seropositive donors. PBMCs from each donor were plated at a concentration of 3 × 10^6 ^cells/ml in a 24 well/plate with 2 ml RPMI complete medium (10% AB human serum, penicillin, gentamycin, glutamine, and 1% HEPES), and directly stimulated with 3 μg/ml of both test and control peptides (1 μg/ml peptides for each 10^6 ^cells). Recombinant human interleukin-2 (rhIL-2, 100 U/ml, PeproTech, Rocky Hill, NJ) was added every other day to the cell culture. At day 15, each batch of cells was washed and directly re-stimulated in fresh medium with either test or control peptide or not re-stimulated. Three hours after re-stimulation cells were harvested to analyze their IFN-γ mRNA transcript production by quantitative real time PCR (qRT-PCR). At three different time points (24, 48, and 72 hours) after re-stimulation supernatants were collected and tested for IFN-γ protein release following the ELISA manufacturer's guidelines.

### Quantitative real time PCR (qRT-PCR)

IFN-γ mRNA transcript production by *in vitro *sensitized PBMCs was evaluated 3 hours after direct peptide re-induction, as previously described [5]. Following 2-week in vitro sensitization (IVS), 2 × 10^5 ^PBMCs (final concentration of 1 × 10^6 ^cells/ml) were plated in a 96 U-bottom well/plate with 200 μl of RPMI complete medium, incubated overnight, and then directly stimulated with 1 μg/ml of specific peptide. After a 3-hour incubation, total RNA was extracted (RNeasy Mini Kit, Qiagen, Valencia, CA) and 1 μl of synthesized cDNA (Invitrogen, Carlsbad, CA) was used as a template to measure IFN-γ mRNA transcription by qRT-PCR using an ABI Prism 7900 Sequence Detection System (Perkin Elmer, Foster City, CA). Quantitative real time PCR results were reported as the number of IFN-γ gene copies normalized by 10^5 ^β-actin gene copies. All PCR assays were performed in triplicate and reported as the average. Stimulation index has been applied based on the negative control values.

### ELISA

The release of IFN-γ protein by *in vitro *sensitized PBMCs after peptide re-stimulation was measured using an enzyme-linked immunosorbent assay (ELISA) kit (Endogen, Woburn, MA). Supernatants of peptide-stimulated PBMCs were collected at 24, 48, and 72 hours. ELISA results were extrapolated from a standard curve generated by linear regression. The assays were performed in duplicate and reported as the average.

## Results

### IFN-γ mRNA transcript production by 9-mer, 10-mer, and 16-mer sensitized PBMCs from HLA-A*2402, A*0101, and Cw*0402 donors re-stimulated with their cognate determinants

**Figure 2:**
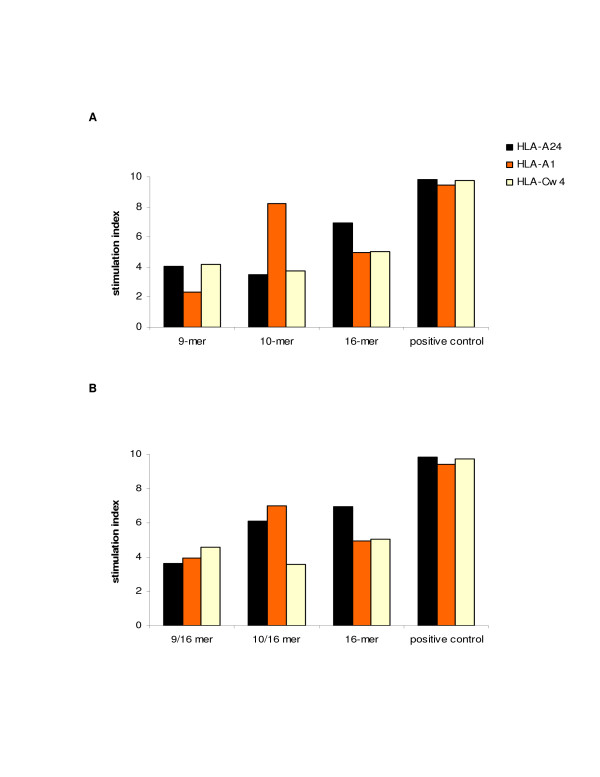
**IFN-γ mRNA transcript production by qRT-PCR. **The ability of the three CMV peptides; pp65_341–349_, pp65_341–350_, and pp65_340–355 _to reactivate an immune T lymphocyte response after their cognate re-stimulation of self-induced PBMCs (A) or after restimulation with each peptide of 16-mer induced PBMCs (B) was analyzed by qRT-PCR. Levels of IFN-γ mRNA transcript were quantitated after a 3-hour peptide induction. The results were performed in triplicate and reported as the average. Levels of IFN-γ mRNA transcript were normalized by levels of β-actin mRNA transcript and the final values were indicated as the ratio over the negative control.

In order to analyze the immediate T cell immune reactivation to each of the three determinants (9-mer, 10-mer and 16-mer) after 2-week in vitro sensitization, qRT-PCR was employed to quantitate levels of IFN-γ mRNA produced by sensitized cells after a 3-hour peptide-induction. Thus, following the 2-week peptide (9-mer, 10-mer, and 16-mer) *in vitro *sensitization, each batch of sensitized cells was re-stimulated with the cognate peptides. Although both 9-mer and 10-mer peptides were able to maintain high levels of stimulation over this time period for all HLA restrictions tested, the 9-mer peptide induced the highest responses in cells expressing HLA-A*2402 (S.I. 4.07–528) or HLA-Cw*0402 (S.I. 4.15–483) while the 10-mer peptide induced the highest responses in cells expressing HLA-A*2402 (S.I. 3.5–528) or HLA-A*0101 (S.I. 8.25–615). The 16-mer peptide was also able to stimulate T cells from all HLA-A*2402, A*0101 and Cw*0402 donors (S.I. 6.95, 4.96, 5.02) at levels that were approximately equal to the average of those induced by each single 9-mer and 10-mer induction (Figure 2A).

### IFN-γ mRNA transcript production by 16-mer peptide-sensitized PBMCs from HLA-A*2402, A*0101, and Cw*0402 donors re-stimulated with either the 9-mer or the 10-mer determinant

In order to analyze the immediate reactivation of memory T lymphocytes following the 16-mer peptide sensitization, the 2-week *in vitro *16-mer peptide sensitized cells were re-stimulated with either 9-mer or 10-mer peptide. Thus, qRT-PCR was employed to quantitate levels of IFN-γ mRNA produced by sensitized cells after a 3-hour peptide-induction. Compared to the previous results, as in figure 2A, that demonstrated the 9-mer peptide's specificity for HLA-A*2402 and HLA-Cw*0402, the re-stimulation of 2-week *in vitro *16-mer peptide sensitized cells with the 9-mer peptide confirmed the 9-mer peptide's specificity for HLA-A*2402 and HLA-Cw*0402 plus its ability to enhance the T lymphocytes reactivity in donors bearing HLA-A*0101 (S.I. 3.96–507). Similarly, the re-stimulation of 2-week *in vitro *16-mer peptide sensitized cells with the 10-mer peptide confirmed this peptide's specificity for HLA-A*0101 while specifically enhancing the T cell reactivity in HLA-A*2402 donors (S.I. 6.12–236). The HLA-Cw*0402 specificity was also confirmed and maintained for the 10-mer peptides (3.58–467), probably adding a new HLA association to this CMV peptide (Figure 2B).

### IFN-γ protein release by 9-mer, 10-mer, and 16-mer peptide-sensitized PBMCs from HLA-A*2402, A*0101, and Cw*0402 donors re-stimulated with their cognate determinants

**Figure 3:**
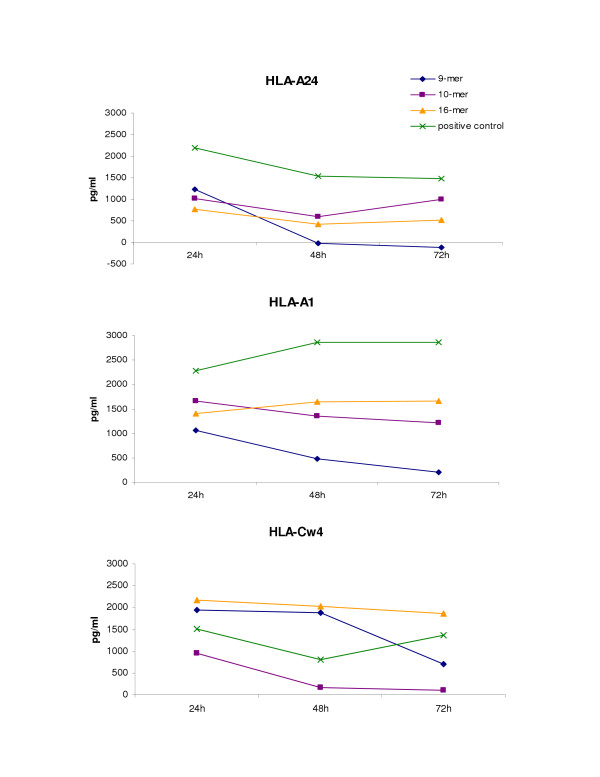
**IFN-γ protein release by ELISA following re-stimulation of peptide (9-mer, 10-mer, and 16-mer) sensitized PBMCs from all donors with their cognate determinants. **Supernatants of peptide (9-mer pp65_341–349_, 10-mer pp65_341–350_, and 16-mer pp65_340–355_) sensitized PBMCs from CMV seropositive donors HLA-A*2402, A*0101, and Cw*0402 were collected at three time point of 24, 48, and 72 hours after cell re-stimulation with each cognate peptide: 9-mer (blue segment), 10-mer (purple segment), and 16-mer (orange segment). The figure shows the peptide induction and the maintenance over time of T lymphocyte reactivation after cognate peptide recall compared to the positive control (green segment) for each individual HLA association: HLA-A24 (top), HLA-A1 (middle), HLA-Cw4 (bottom).

Following 2-week *in vitro *sensitization with 9-mer, 10-mer or 16-mer peptides of PBMCs from HLA-A*2402, A*0101, and Cw*0402 donors, supernatants were collected from cell cultures at 24, 48 and 72 hours after the re-stimulation of each sensitized batch of cells with the cognate peptides. Despite the fact that both the 9-mer and 10-mer peptides were able to reactivate cells from each of the three donors at time 0 (Figure 2A), the 9-mer peptide was weaker than the 10-mer peptide in maintaining a consistent immune T response over time in both the HLA-A*2402 and HLA-A*0101 donors. In contrast, the 9-mer peptide was stronger than the 10-mer in stimulating and maintaining a T immune reactivation and response over time in donors bearing HLA-Cw*0402. The stimulation with the 16-mer peptide induced and maintained consistent immune T cell reactivation in all donors tested, as assessed by levels of IFN-γ protein production when compared to the positive control (Figure 3).

### IFN-γ protein release by 16-mer sensitized PBMCs from HLA-A*2402, A*0101, and Cw*0402 donors re-stimulated with either 9-mer or 10-mer determinant

**Figure 4:**
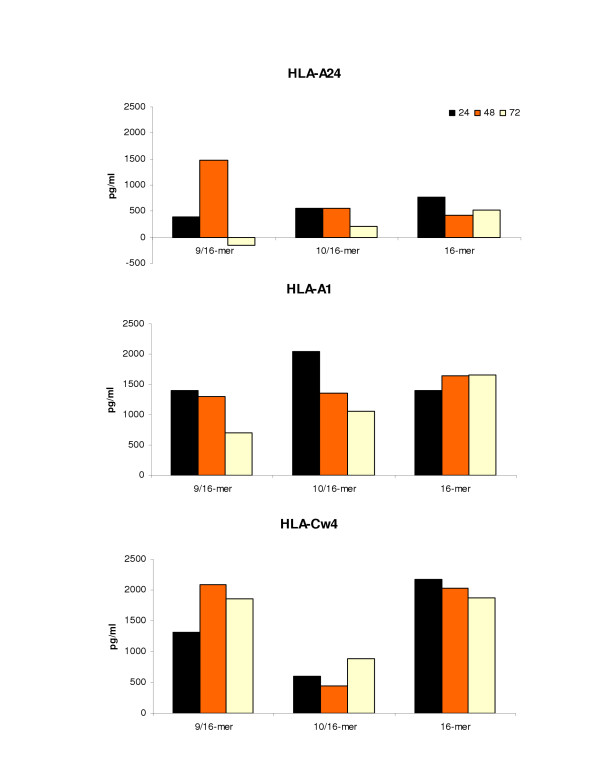
**IFN-γ protein release ELISA following re-stimulated of 16-mer sensitized PBMCs from all donors with either the 9-mer or 10-mer determinant. **Supernatants of 16-mer pp65_340–355_-sensitized PBMCs from CMV seropositive donors HLA-A*2402, A*0101, and Cw*0402 were collected at the three time points of 24 hours (black), 48 hours (orange), and 72 hours (yellow) after cell re-stimulation with either the 9-mer pp65_341–349 _or 10-mer pp65_341–350 _peptide. The figure shows the peptide induction and maintenance over time of the T lymphocyte reactivation from 16-mer pp65_340–355_-sensitized PBMCs after either 9-mer pp65 _341–349 _(9/16) or 10-mer pp65 _341–350 _(10/16) peptide recall compared to the specific 16-mer pp65_340–355 _re-induction (16) for each individual HLA association: HLA-A24 (top), HLA-A1 (middle), HLA-Cw4 (bottom).

Following 2-week *in vitro *sensitization of PBMCs from HLA-A*2402, A*0101, and Cw*0402 donors with the 16-mer sequence, supernatants were collected from cell culture at 24, 48 and 72 hours after re-stimulation of 16-mer peptide-sensitized cells with either 9-mer or 10-mer peptides. It seems that in all donors the re-stimulation of 16-mer *in vitro *sensitized cells with the 9-mer and the 10-mer peptides was able to better induce IFN-γ protein production than 9-mer or 10-mer *in vitro *sensitized cells that were stimulated with the cognate peptide either by enhancing cytokine protein production at each time point or by better maintaining cytokine protein release over time. Specifically, this was seen in both HLA-A*2402 and HLA-A*0101 donors following 9-mer peptide induction and in the HLA-Cw*0402 donor following 10-mer peptide induction (Figure 4).

## Discussion

The adoptive transfer of immunodominant T lymphocytes to CMV-infected transplanted patients represents one of the treatments of choice to clear the CMV disease and to quickly reconstitute the lost balance between the CMV infection and the immune system [10-12]. The use of specific immune peptides to detect and expand immunocompetent T cells is an important tool that has been already applied in several clinical trials [13,14]. Once CMV epitope mapping had been initiated and led to the identification of epitopes encompassing several HLA class I antigens, great effots were devoted to the identification of HLA cross-reactivity of known immune determinants and the identification of new determinants. In this specific case, the two overlapping peptides pp65_341–349 _(QYDPVAALF) and pp65_341–350 _(QYDPVAALFF) have been shown to have a marked cross-reactivity. First, it was shown that pp65_341–349 _is restricted to HLA-A*2402 and Cw*0402 and pp65_341–350 _to HLA-A*2402 and A*0101 [6,7]. In this study a new HLA specificity for the 9-mer to HLA-A*0101 and for the 10-mer to HLA-Cw*0402 is well described. Furthermore, this study showed that both peptides were able to effectively stimulate immune T cell responses from HLA-A*2402, A*0101 and Cw*0402 donors, confirming that these peptides are promiscuous and not restricted to a single HLA type. While both the 9-mer and 10-mer peptides are able to maintain high levels of stimulation over time in HLA-A*2402 donors, both are also able to induce and maintain an immune reactivation in donors bearing their other noted HLA associations. In fact, the 9-mer is better able to maintain stimulation in HLA-Cw*0402 donors and the 10-mer is better able to do so in HLA-A*0101 donors.

Interestingly, another new finding emerged: both the 9-mer and 10-mer peptides were able to induce a population of restricted T lymphocytes from a cell population that had been *in vitro *sensitized with a peptide sequence that encompassed the two peptides. The 16-mer peptide is able to effectively stimulate T cells from HLA-A*2402, A*0101 and Cw*0402 at levels that are well maintained over time. In particular, it seems that the 9-mer and 10-mer peptide reactivation of cells previously sensitized with the 16-mer peptide results in enhanced T cell reactivation in all donors. Although these results need to be confirmed and validated by further *in vivo *studies, we speculate that the use of this 16-mer region rather than the single 9-mer or 10-mer peptides would be advantageous in clinical modalities such as adoptive transfer of epitope-specific T lymphocytes or epitope-specific vaccinations. These results support the potential use of the 16-mer peptide in CMV adoptive immune therapy [15].

## Supplementary Material

Additional File 1 Table 1. to go DOC Click here for file
