# Supplementary material for: Analysis of memory T lymphocyte activity following stimulation with overlapping HLA-A*2402, A*0101 and Cw*0402 restricted CMV pp65 peptides
Source: J Transl Med. 2005 May 26;3:23. doi: 10.1186/1479-5876-3-23 (PMC1175859; doi:10.1186/1479-5876-3-23)
Supplement: Additional File 1 — Table 1. to go DOC [file 1479-5876-3-23-S1.doc]

**Length Peptide Sequence __________**

20-mer pp65_340-359_ N-RQYDPVAALFFFDIDLLLQR-C

16-mer pp65_340-355_ N-RQYDPVAALFFFDIDL---------C

The original 20-mer sequence was reduced to 16-mer sequence by C- terminus cleavage in order to allow its reconstitution. The C-terminus reduction (N-LLQR-C) did not affect the aim of having both 9-mer (QYDPVAALF, pp65_341-349_) and 10-mer (QYDPVAALFF, pp65_341-350_) represented in the sequence.
